# The influence of the choroid plexus on brain function: beyond its role in cerebrospinal fluid production

**DOI:** 10.1186/s41232-025-00386-1

**Published:** 2025-07-02

**Authors:** Sayako Katada, Kelren S. Rodrigues, Kinichi Nakashima

**Affiliations:** Department of Stem Cell Biology and Medicine, Graduate School of Medical Sciences, Kyushu University, 3-1-1 Maidashi, Higashi-Ku, Fukuoka, 812-8582 Japan

**Keywords:** Choroid plexus, Cerebrospinal fluid, Blood-CSF barrier, Neuroinflammation

## Abstract

The choroid plexus (ChP) is a highly vascularized tissue located within the brain ventricles. Traditionally recognized for its primary role in cerebrospinal fluid (CSF) production, recent research has unveiled a far more complex and dynamic picture of the ChP’s contributions to brain health and homeostasis. The ChP is composed of tight-junction-bound epithelial cells and the underlying stroma-rich fenestrated capillaries of blood vessels. This unique architecture creates a barrier between the peripheral blood and CSF, regulating the brain’s internal environment. The discovery that CSF enters the brain parenchyma via the perivascular space, coupled with the identification of a functional brain lymphatic system linked to CSF turnover, further highlights the ChP as a gatekeeper of waste clearance and fluid homeostasis. This review will cover the development and histology of ChP, focusing on the dynamic response of the blood-CSF barrier in the context of systemic inflammation, a process whose molecular mechanisms have recently been elucidated.

## Introduction

The choroid plexus (ChP) is a sheet of organs, made up of blood vessels and specialized epithelial cells, located in the roof of the two lateral ventricles and of the third and fourth ventricles of the brain. It is responsible for producing the majority of cerebrospinal fluid (CSF) that circulates throughout the ventricular system, bathing the brain and spinal cord [1]. CSF flows from the fourth ventricle into the subarachnoid space, where it exits the intracranial cavity via meningeal lymphatic vessels and through lymphatic drainage along cranial and spinal nerves.

The most extensively studied function of ChP is its vital role in maintaining the blood-CSF barrier. For decades, the brain was considered an “immune privileged” organ, believed to lack interaction with the peripheral immune system [2]. However, the discovery that during systemic inflammation, activated T cells and macrophages could penetrate the blood-CSF barrier and enter the brain parenchyma, cast doubt on this theory at the close of the twentieth century [3, 4]. As an interface between the central nervous system (CNS) and a vascularized stroma that is home to a variety of immune cells, the ChP serves as a selective gateway as well as a protective barrier against infectious agents [5, 6]. This dual function allows for the controlled entry of specific cells and molecules essential for maintaining brain homeostasis.

Herein, while outlining the distinct morphological and functional characteristics of ChP in each brain ventricle, we focus on the role of the blood–CSF barrier that has been intensively studied recently. We especially delve into its emerging roles in brain immune surveillance and healing properties, highlighting the ChP’s multifaceted contributions to brain homeostasis.

## Development of the ChP: a vital role in cortical formation

**Fig. 1:**
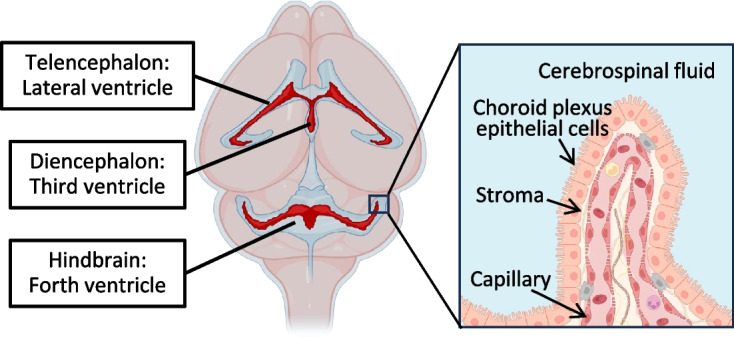
The location and structure of the choroid plexuses. Schematic diagram showing the location of the lateral, third and fourth ventricular choroid plexus, depicted with a simplified illustration. The choroid plexus consists of an outer layer of cuboidal epithelial cells surrounding a core of capillaries and stromal cells. It forms the blood-CSF barrier and serves as a gateway for immune cells to enter the CNS

ChP epithelial cells originate from the same neuroepithelium as multipotent stem cells that give rise to the nervous system, whereas the stromal component of the ChP is believed to arise from brain mesenchymal cells [1]. The appearance of ChPs in different ventricles is slightly different in developmental times, with the hindbrain ChP in the fourth ventricle being the first to become visible around E9.5 in mice, followed by the telencephalic ChP in the lateral and diencephalic ChP in the third ventricles [1] (Fig. 1). Gene expression analyses have revealed that each ChP retains a developmental blueprint of its origins along the body axis, with ventricle-specific transcriptomes for lateral, third, and fourth ventricle ChP. For example, the ChP of the lateral ventricle is enriched for markers of the posterior half of the telencephalon, such as Empty spiracles homeobox 2 (Emx2), Orthodenticle homeobox1 (Otx1), and Six homeobox 3 (Six3) [7]. In contrast, the ChP in the third or fourth ventricles is enriched in Solute carrier family 4a2 (Slc4a2) and R-spondin 3 (Rspo3), or Homeobox A2 (HoxA2), Meis homeobox 1 (Meis1), and Wnt ligand secretion mediator (Wls), respectively [8]. Since the expression of these transcription factors differs slightly in each choroid plexus, the expression of genes encoding secreted proteins may also differ from ventricle to ventricle, contributing to the regionalized CSF production. Indeed, differences in ChP protein secretion between the lateral and fourth ventricle ChPs have been well analyzed by secretome and transcriptome analyses, with embryonic ChPs showing robust local gene expression that decreases with age in a gene-specific manner [7]. The expression and secretion of Sonic Hedgehog (Shh) are dominant in the ChP of the fourth ventricle. The Shh signaling is known to promote pericyte-mediated vascular outgrowth and stimulates the proliferation of hindbrain ChP progenitors, which are located adjacent to the lower rhombic lip [9, 10]. On the other hand, the ChP in the lateral ventricle secretes a higher amount of cystatin C and cathepsin B/D, compared with that in the fourth ventricle [7]. Given the function of these enzymes in axonal pruning and dendritic spine morphogenesis, differences in their mode of secretion could account for the different properties of telencephalic and hindbrain neurons. In the fetal brain, the ChP is the major source of Insulin-like growth factor (Igf2) which provides essential growth and survival-promoting factors for neural stem cells (NSCs) [11]. Notably, the peak levels of Igf2 expression in the ChP and the amount in CSF during late gestation coincides with a period of intense corticogenesis in rodents [11]. Orthodenticle homeobox 2 (Otx2), likely secreted from the ChP in the lateral ventricle and distributed throughout the brain via the CSF, is taken up by parvalbumin-positive GABAergic interneurons in the forebrain. This uptake is hypothesized to regulate interneuron maturation, consequently influencing the timing of the critical period for binocular vision plasticity [12]. Upon NSCs differentiating into neuroblasts, Slit family proteins in the CSF act as chemo-repulsive cues, directing neuroblasts along the rostral migratory stream toward the olfactory bulb [13]. Consequently, the development of the mammalian brain hinges on precise interactions between multiple signaling molecules that are transported to neural cells through the CSF.

## Morphological features of ChP specialized for CSF production

**Fig. 2:**
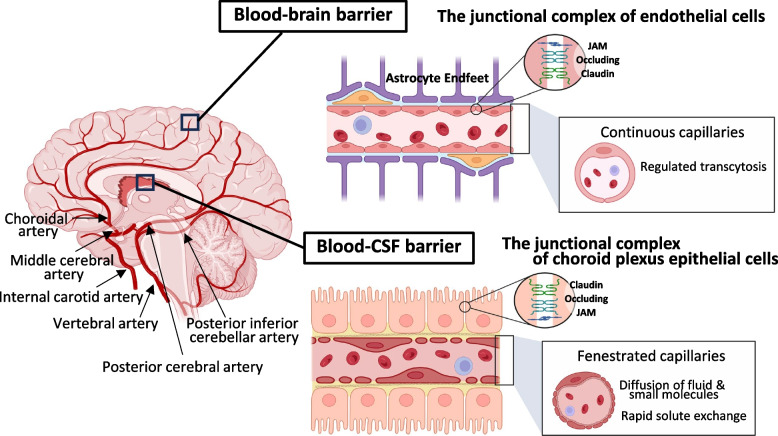
Schematic illustration of brain arteries and representation of the blood-brain and blood-CSF barrier. Brain capillaries are characterized by continuous endothelium with low transcellular transport. These capillaries consist of a monolayer of polarized endothelial cells interconnected by tight junctions and are further ensheathed by astrocytic endfeet, forming a robust blood-brain barrier. In contrast, the capillaries of the ChP are fenestrated and not connected by tight junctions, allowing for the rapid passage of small molecules and water. Therefore, the associated choroid plexus epithelial cells and their tight junction components form the blood-CSF barrier

Despite sharing a common gross structure, each ChP exhibits significant variations in compactness and size. The third ventricular ChP is the smallest, while the fourth is the most compact, and the lateral demonstrates the most expanded structure spreading throughout the ventricles [1]. The blood supply to the ChP varies depending on its location. The telencephalic ChP receives blood from the anterior choroidal artery, which branches from the internal carotid or middle cerebral artery. The posterior choroidal artery, originating from the posterior cerebral artery, supplies both the telencephalic and diencephalic ChP. Finally, the hindbrain ChP is vascularized by the anterior and posterior inferior cerebellar arteries, arising from the basilar and vertebral arteries [5, 14]. Blood flow and CSF secretion are partially regulated by sympathetic and parasympathetic innervation [15]. In the brain parenchyma, the endothelium of continuous capillaries exhibits extremely low rates of transcellular vascular transport. These endothelium are connected by tight junction components such as claudin-5, occludin, and junctional adhesion molecules (JAMs), along with the additional coverage provided by astrocytic endfeet, creating a robust blood–brain barrier (Fig. 2). However, the capillaries of the ChP are fenestrated, interconnected by thin diaphragms that allow for the rapid passage of small molecules and water [5, 16]. This facilitates the swift delivery of water from the bloodstream to epithelial cells for CSF production, and the tight junctions between the ChP epithelial cells are the key component of the blood-CSF barrier.

The ChP exhibits a structure optimized for its secretory function. It comprises a monolayer of epithelial cells encasing a core of capillaries and connective tissue. Tight junctions seal adjacent ChP epithelial cells, forming the blood-CSF barrier, which restricts the paracellular diffusion of molecules from the systemic circulation into CSF. Along with adherens junctions, these junctions maintain the apical-basal polarity of important membrane proteins such as channels and transporters [5]. Evidence from embryonic ChP, indicating the presence of junctional and extracellular matrix proteins, suggests the establishment of early barrier functions [17]. While studies using injectable tracers support the integrity of these barriers early in development, transcriptome analyses reveal dynamic gene expression changes related to transporters and channels, indicating that the blood-CSF barrier undergoes dramatic changes during development [18, 19]. This likely reflects the differential regulation of CSF production between embryonic and mature brains [8, 20].

## The ChP is a site of immune cell infiltration into the brain

**Fig. 3:**
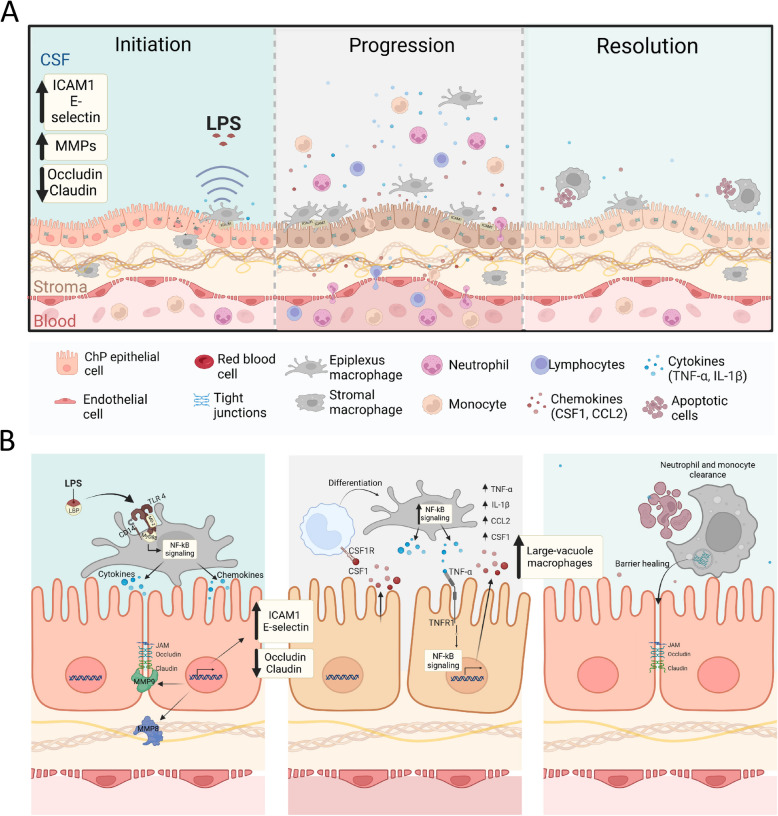
The dynamic response of the ChP over time in a systemic inflammation model. **A** General framework of inflammatory response. *Initiation*: upon LPS detection, the immune system is activated, leading to the downregulation of molecules essential for barrier integrity and upregulation of immunomodulatory genes; *Progression*: the ChP displayed a heightened inflammatory state, marked by increased cytokine/chemokine production and immune cell infiltration; *Resolution*: barrier healing and the removal of infiltrated immune cells. **B** Dynamic signaling interplay between macrophages and ChP epithelial cells during inflammatory processes. *Initiation*: LPS detection by epiplexus macrophages initiates TLR4/MyD88/NF-κB signaling, which leads to pro-inflammatory cytokine release and basal lamina disruption; *Progression*: macrophage-secreted TNF-α and IL-1β activate TNFR1/NF-κB signaling in ChP epithelial cells, inducing CSF1 production, which supports monocyte differentiation and macrophage survival; *Resolution*: macrophages with large vacuoles are more prevalent and contribute to neutrophil clearance and tight junction repair, via Occludin internalization and potential recycling, ultimately restoring the ChP’s barrier

A growing body of research has recently demonstrated interest in the ChP and its role in brain immunity, emphasizing how it controls immune cell entry into the brain [5, 21]. Various models of systemic inflammation have been described, emphasizing the key role of the ChP in infections and pathogen invasion [22]. The most widely used model is the administration of lipopolysaccharide (LPS), a component of Gram-negative bacteria, known to increase the presence of peripheral immune cells in the ChP and CSF [23–25]. Here, we primarily focus on the LPS exposure model as a key example to illustrate how systemic inflammation impacts the ChP. We will provide a comprehensive overview of the ChP’s temporal response to inflammation, encompassing initial immune activation, the progression of inflammation, and the final healing phase marked by restoration of the ChP barrier (Fig. 3).

### Initial activation of the ChP immune response

ChP stroma contains a diverse population of immune cells, including myeloid cells, such as dendritic cells and macrophages, as well as blood-derived innate immune cells [8, 26]. This unique immune environment enables the ChP to rapidly respond to potential threats to brain homeostasis.

Lipopolysaccharide coupled with LPS-binding protein (LBP) binds to CD14 on the cell membrane, facilitating its transfer to the Toll-like receptor 4 (Tlr4)/myeloid differentiation factor 2 (MD-2) complex [24, 27]. This interaction activates the MyD88/NF-κB and mitogen-activated protein kinase (MAPK) signaling pathways, which are crucial for initiating both immune responses and changes ChP’s barrier activity [27, 28]. Within the initial hours following LPS injection, an increased number of neutrophils can be observed in the ChP stroma and on the apical surface facing the CSF, accompanied by disruptions in epithelial tight junctions [29]. Transcriptional profiling of the ChP in a mouse model following LPS exposure revealed that a subset of downregulated genes were associated with maintenance of barrier integrity, while upregulated genes were primarily involved in immune modulation [24]. Specifically, ChP epithelial cells upregulate pro-inflammatory signaling molecules (Tlr1-4), which correlates with reduced expression of Occludin, a tight junction protein crucial for blood-CSF barrier [30, 31]. In the meantime, NF-κB activation increases matrix metalloproteinases (MMPs) production. MMP9 degrades key barrier proteins like Claudin-5 [32], while MMP8 alters the basal lamina by cleaving structural components like collagen I, compromising barrier integrity and epithelial morphology [33]. Following an initial increase in neutrophils, monocytes and macrophages become predominant in the CSF, ChP stroma, and epiplexus regions [29, 34]. This recruitment is facilitated by the expression of adhesion molecules, such as E-selectin, on endothelial cells. E-selectin expression is also known to be induced by cytokines like Interleukin-1β (IL-1β) and tumor necrosis factor-α (TNF-α) [35, 36] and plays a crucial role in the rolling phase of leukocyte extravasation from the bloodstream into surrounding tissues [37].

### Dynamics of choroid plexus during inflammation progression

Resident macrophages, which are distributed throughout the ChP, have recently received significant attention regarding inflammation progression [29, 38]. A recent study by Xu et al. utilized single-cell RNA-sequence (scRNA-seq) analysis to show that resident macrophages in the ChP launch a strong inflammatory response within 24 h of LPS injection [39]. This response included increased production of inflammatory chemokines like Ccl and Cxcl family members, highlighting the active role of ChP macrophages in combating inflammation in the brain. The fact that this reaction was temporary and returned to normal levels within 72 h emphasizes how these macrophages play a part in starting the leukocyte recruitment process during acute inflammation [39]. The authors also highlight the emergence of a specific Chil1(+), and Icam1(+) state of epithelial cells at 24 h post-LPS exposure [39]. The production of matrix remodeling factors was significantly elevated in these cells, which helps to break down tight junctions that occur concurrently with immune cell infiltration into the ChP.

TNF-α, a key mediator of the inflammatory cascade secreted by macrophages during inflammation, has been demonstrated to increase the expression of Intercellular adhesion molecule 1 (Icam1) and Vascular cell adhesion molecule 1 (Vcam1) in ChP epithelial cells [40]. These adhesion molecules are critical for immune cell recruitment and retention. Furthermore, Wang et al. also observed that the simultaneous application of LPS and TNF-α increases NF-κB phosphorylation, along with an increased activation of TNFR1-NF-κB signaling in ChP epithelial cells [41]. NF-κB activation in ChP epithelial cells, in turn, promotes the expression of TNF-α and IL-1β, potentially influencing Colony-stimulating factor 1 (CSF1) production [42]. A crucial component of monocyte differentiation and macrophage survival, CSF1 is further expressed by epithelial cells in response to this signaling activation [43]. The temporal dynamics of CSF1 expression in epithelial cells are correlated with monocyte infiltration 24 h post-LPS injection, and their subsequent differentiation into macrophages and dendritic cells in a later phase [39]. This coordinated action demonstrates how intricately immune components and epithelial cells interact to maintain the inflammatory response and modify barrier function.

Notably, this dynamic interaction between macrophages and ChP epithelial cells is not restricted to the LPS systemic immune activation. A study by Vlaminck et al. using a mouse model of *Trypanosoma brucei* infection (a parasitic brain invasion) further demonstrates this complex interplay. They show how resident and recruited macrophages orchestrate the immune response to invading parasites across brain barriers [44]. Furthermore, in vivo two-photon imaging in the maternal immune activation (MIA) mouse model demonstrated that ChP-resident macrophages, along with their surveillance function, are present during embryogenesis [45]. Following MIA, elevated levels of pro-inflammatory cytokines, particularly CCL2, actively recruited immune cells to enter the CSF through specific infiltration points in the ChP villi. Using viral transduction, the selective expression of CCL2 in the ChP demonstrated increased recruitment of CCR2-expressing cells during inflammation, together with enhanced macrophage motility in the developing ChP, indicating active surveillance behavior. Furthermore, as observed after MIA, increased CSF-CCL2 levels were sufficient to disrupt the expression and/or subcellular organization of key molecules (ZO-1, Occludin, and Claudin-1) essential for maintaining ChP barrier function [45].

### Resolution of inflammation and subsequent ChP barrier healing

As discussed in the previous sections, the activated inflammatory response within the ChP epithelial and residential cells leads to dramatic changes in cellular composition, gene expression, and the integrity of the blood-CSF barrier. This raises the question of how ChP-associated cells regulate this environment and return ChP to its baseline physiological state. Recent findings suggest that macrophages, beyond their role in sustaining inflammation, also play a critical role in repairing tight junctions and clearing neutrophils, thereby contributing to the restoration of ChP barrier function [39]. It was observed that by 48 h following LPS exposure, there were significant increases in the number, motility, and mobility of epiplexus-resident macrophages, as well as the appearance of large vacuoles [39]. Their vacuole content analysis revealed the presence of Ly6G/C + monocytes or neutrophils. Notably, scRNA-seq has revealed that Occludin, a key component of tight junctions, was identified within these macrophages [39]. This implies that in addition to eliminating damaged neutrophils, macrophages actively take part in tight junction repair by internalizing and potentially recycling Occludin, which helps restore the integrity of the ChP barrier. It is interesting to note that, in addition to the blood-derived monocyte population, macrophage-marker-positive cells were also found moving from the CSF to the ChP after the infection progression. These CSF-derived macrophages exhibited unique characteristics, lacking both microglia and barrier-associated macrophage signature genes [39]. It is possible that these macrophages can complement ChP repair, despite the precise origin and function of this unique population are still unknown. This would further support the multifaceted role of macrophages in resolving neuroinflammation and reestablishing barrier function.

In support of these findings, Vlaminck et al. showed that brain barrier integrity is quickly restored after Melarsoprol (an antiprotozoal agent) treatment using a *Trypanosoma brucei* infection model [44]. Myeloid cells that had infiltrated the brain and its border regions quickly disappeared during the resolution phase. The study revealed that recruited macrophages are short-lived and are quickly removed from the brain once the infection resolves [44]. However, further research is needed to fully elucidate the precise mechanisms underlying the activation of these resident macrophages and their role in eliminating infiltrated cells.

## Conclusion and prospective

In recent years, the production of CSF has emerged as a significant, yet largely unexplored, factor that may influence glymphatic CSF transport within the CNS. The glymphatic system is a macroscopic waste clearance system that utilizes a unique system of perivascular channels formed by astrocytes to facilitate the efficient elimination of soluble proteins and metabolites from the CNS. The name “glymphatic” was coined to reflect the system dependence on glial cells (astrocytes) and its functional similarity to the lymphatic system [46]. Interestingly, the glymphatic system functions primarily during sleep, with little function during wakefulness [47]. Thus, the universal need for sleep across species may reflect the brain’s requirement to actively eliminate potentially neurotoxic waste products, such as amyloid-β. Indeed, chronic non-infectious diseases such as Alzheimer’s disease and multiple sclerosis, which often cause persistent inflammation in the CNS, have been reported to have decreased CSF production [22]. Hence, there is significant interest in improving or manipulating the ability to produce CSF in these diseases. The ChP, despite its small size, exerts influence far beyond a simple secretory organ due to its diverse functions. Thus, there are plenty of reasons for a continued strong interest in ChP from the point of view of the immunologist, neuropathologist as well as neurodevelopmental biologist.

## Data Availability

Not applicable.
